# Uncommon presentation of left main congenital coronary aneurysm: a rare case report

**DOI:** 10.3389/fcvm.2024.1504835

**Published:** 2024-12-02

**Authors:** Peng Xu, Shili Zhong, Hui Tan

**Affiliations:** ^1^ Rehabilitation Medicine Department, Army Medical Center of PLA, Chongqing, China; ^2^ Department of Intensive Care Medicine, Army Medical Center of PLA, Chongqing, China; ^3^ Health Management Center, The Third Affiliated Hospital of Chongqing Medical University, Chongqing, China

**Keywords:** coronary aneurysm, conservative treatment, coronary angiography, myocarditis, interventional therapy

## Abstract

Coronary aneurysm, a dilated segment of the coronary artery, is a rare condition with a prevalence ranging from 0.02% to 0.2%. According to the current literature, reports of large aneurysms in the left main artery are extremely rare. We present a case of a 43-year-old male patient presenting with cough, wheezing, and dyspnea after a cold. Initial examinations suggested viral myocarditis, but further evaluation revealed a giant aneurysm in the left main coronary artery. Due to the high surgical risk, conservative treatment was chosen. Follow-up assessments showed no significant changes in the coronary aneurysm, with slight improvement in dyspnea. This rare case of a left main congenital coronary aneurysm suggests that treatment should consider the patient's overall condition, thrombosis presence, suitability for anticoagulant therapy, and aneurysm location and size.

## 1 Background

Coronary angioma is characterized as a dilated segment of the coronary artery that exceeds 1.5 times the diameter of adjacent normal segments (1). Based on current literature, the prevalence of large coronary aneurysms ranges from 0.02% to 0.2% (2). However, the prevalence of large coronary aneurysms measuring 5 cm or more in diameter is even lower, less than 0.02% (1–3). The right coronary artery is the most commonly affected, accounting for 40%–70% of cases, followed by the circumferential artery (23%) and the anterior descending branch (32%) (1, 2, 4). Involvement of all three vessels or the left coronary trunk is rare.

## 2 Case presentation

This case report presents the clinical details of a 43-year-old male patient who developed a cough and sneezing following a recent cold. Although the cold symptoms improved with self-administered medication, the patient experienced wheezing and paroxysmal dyspnea after physical activity. An electrocardiography(ECG) at a local hospital revealed rapid ventricular rate atrial fibrillation with variable conduction.

**Figure 1:**
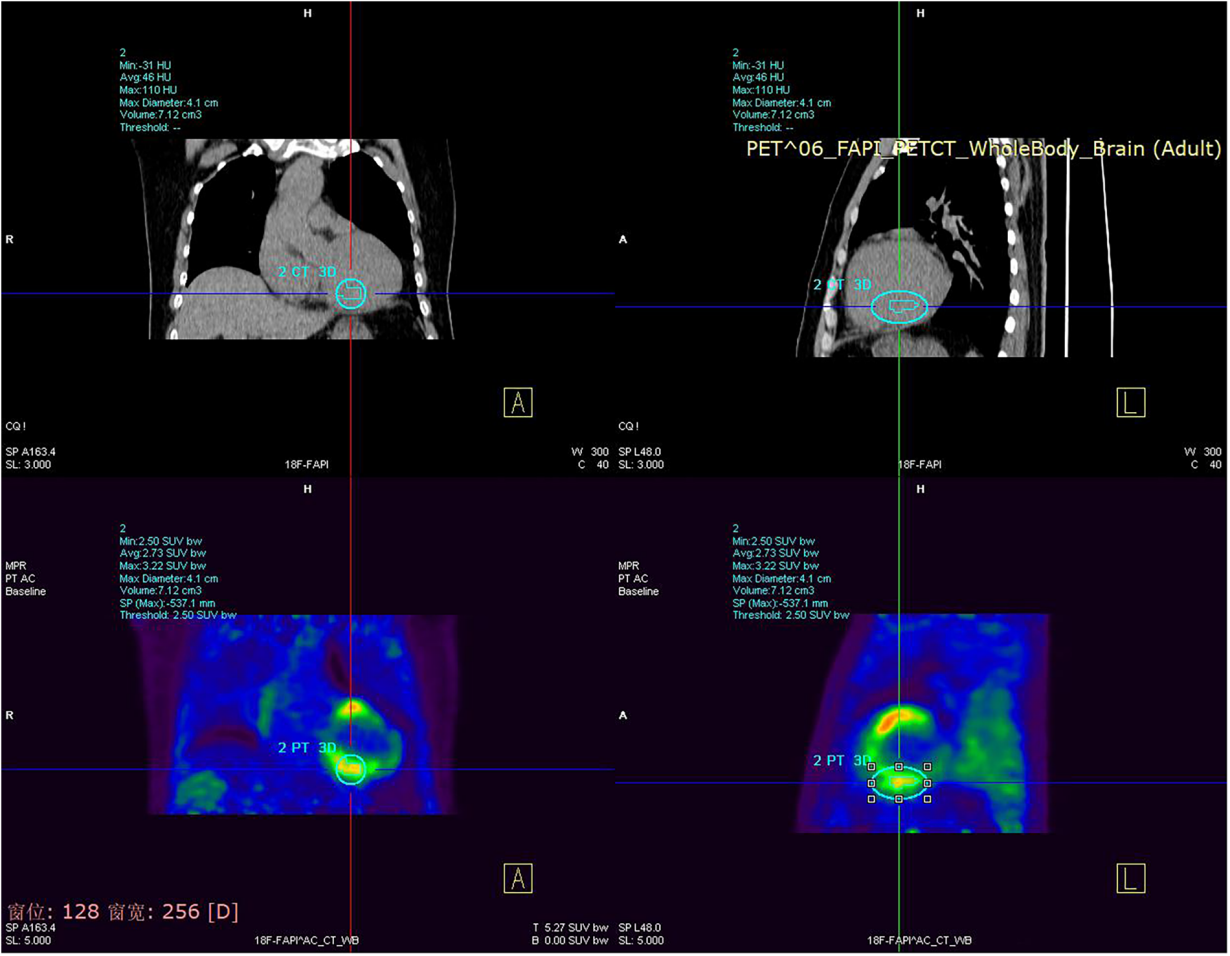
Myocardial nuclide imaging (PET) revealed increased FAPI uptake throughout the left ventricle and right atrium.

**Figure 2:**
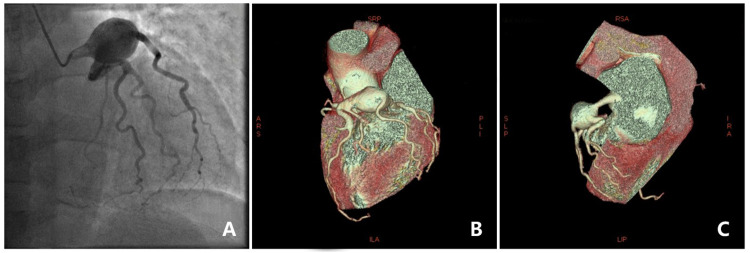
Coronary angiography image and 3D reconstruction. **(A)** Coronary angiography showed a large aneurysm. **(B,C)** 3D reconstruction of an aneurysm in a coronary artery.

The cardiac ultrasound indicated that the LVEF was 38% and the left atrium and ventricle were enlargement with weakened ventricular septal motion. Moderate mitral and mild aortic and pulmonary regurgitation were also noted. The patient also displayed a troponin sensitivity of 0.023 ug/L. Based on these findings, the local hospital suspected viral myocarditis. The patient was referred to our cardiovascular department for further assessment. The patient's NT pro-BNP level was measured at 1,169 pg/ml, and myocardial nuclide imaging (PET) revealed increased fibroblast activation protein inhibitors(FAPI) uptake throughout the left ventricle and right atrium (Figure 1), supporting a diagnosis of myocarditis. The patient received symptomatic treatment, including hormone anti-inflammatory therapy, nutritional support for myocarditis, and gamma globulin therapy. However, these interventions provided limited relief from wheezing. Coronary angiography showed a left coronary dominant type, with no obvious stenosis in the left main trunk but a 2.7 × 2.3 cm aneurysm in the distal part of the left main trunk. No stenosis was observed in the anterior descending branch, circumflex branch, or right coronary artery (Figure 2). Autoimmune workup, including ANA, anti-dsDNA, ENA antibody profile, and ANCA, returned negative results, ruling out autoimmune causes of the aneurysm. Given the giant aneurysm in the left main trunk, myocarditis, and heart failure with intermediate ejection fraction, open-heart surgery with cardiopulmonary bypass, partial tumor resection, and coronary artery bypass grafting was recommended. However, due to the high risk, the patient and family opted for conservative treatment. As intravascular ultrasound (IVUS) was not performed during coronary angiography, antithrombotic or anticoagulant therapy was not initiated. Instead, the patient was prescribed atorvastatin 20 mg nightly and metoprolol succinate sustained-release 47.5 mg daily.

The patient was closely monitored at 3, 6, 9, and 12 months post-discharge. During follow-up, the coronary aneurysm showed no significant changes, and the patient reported slight improvement in dyspnea, though other symptoms and physical signs remained stable.

## 3 Discussion

The exact cause of coronary aneurysm formation remains unclear. However, studies have shown that matrix metalloproteinases play a role in the pathogenesis of CAA formation by increasing the breakdown of proteins in the extracellular matrix (5, 6). These enzymes have the ability to degrade various components of the arterial wall matrix and are found in higher concentrations in aneurysms. There is no consensus on the definition of a giant coronary aneurysm. In most literature, it is considered a giant coronary aneurysm if the dilation of the coronary artery is more than 1.5 times the diameter of adjacent normal reference vessels or if the vessel diameter is directly greater than 20 mm (7–9).

Coronary aneurysms can have several etiologies, including atherosclerosis, autoimmune diseases, and Kawasaki disease (9, 10). In this case, the patient's autoimmune screening and coronary angiography findings were negative, suggesting a high likelihood of congenital origin. Coronary aneurysms can present with a range of clinical manifestations, such as asymptomatic chest pain, chest tightness, and pericardial tamponade (11). Initially, the patient did not experience any symptoms but was admitted to the hospital this time due to wheezing and dyspnea after catching a cold. Myocardial nuclide imaging was performed to consider myocarditis, but the symptoms did not improve significantly with treatment. Subsequent coronary angiography revealed the presence of a large coronary aneurysm.

Thrombus formation occurs in coronary aneurysms due to slow blood flow and platelet activation. To better assess aneurysm condition, intravascular ultrasound (IVUS) is recommended when a coronary aneurysm is detected via angiography. In the case with thrombosis, coagulation function, thromboelastography (TEG), and platelet function tests are advised. With timely anticoagulation or antithrombotic therapyare necessary. According to a 2021 study by Tuncay Taskesen, patients without symptoms, coronary occlusion, or thrombosis may not require specialized treatment (12). Additionally, Cihan Ozturk reported a case of an asymptomatic patient with a right coronary aneurysm who remained symptom-free for many years (13). However, for patients with obstructive coronary artery disease symptoms or myocardial ischemia due to embolization, surgical intervention is appropriate. Surgical options include interventional therapy, aneurysm excision, ligation, or coronary artery bypass surgery (2). In a notable 2024 case report by Najdat Bazarbashi, a patient with a large coronary aneurysm was successfully treated with a combination of stent placement and coil embolization (14). For cases involving multiple vessel disease, left main coronary artery aneurysm, or complications like fistula, compression, or rupture, surgical treatment is typically indicated. However, our patient opted for conservative management, which led to symptom alleviation over follow-up. If symptoms worsen, surgical or interventional treatments may become necessary, though surgical costs are high, and clinical evidence on specific prognostic outcomes is limited.

## 4 Conclusion

Left main congenital coronary aneurysms are exceedingly rare. Proper diagnosis and treatment require assessment of thrombus presence within the aneurysm, along with evaluation of coagulation and platelet function to guide anticoagulation or antiplatelet therapy. For surgical decision-making, factors such as aneurysm location, size, arterial wall condition, and overall patient health should be carefully considered to select an appropriate treatment approach.

## Data Availability

The original contributions presented in the study are included in the article/Supplementary Material, further inquiries can be directed to the corresponding author.
